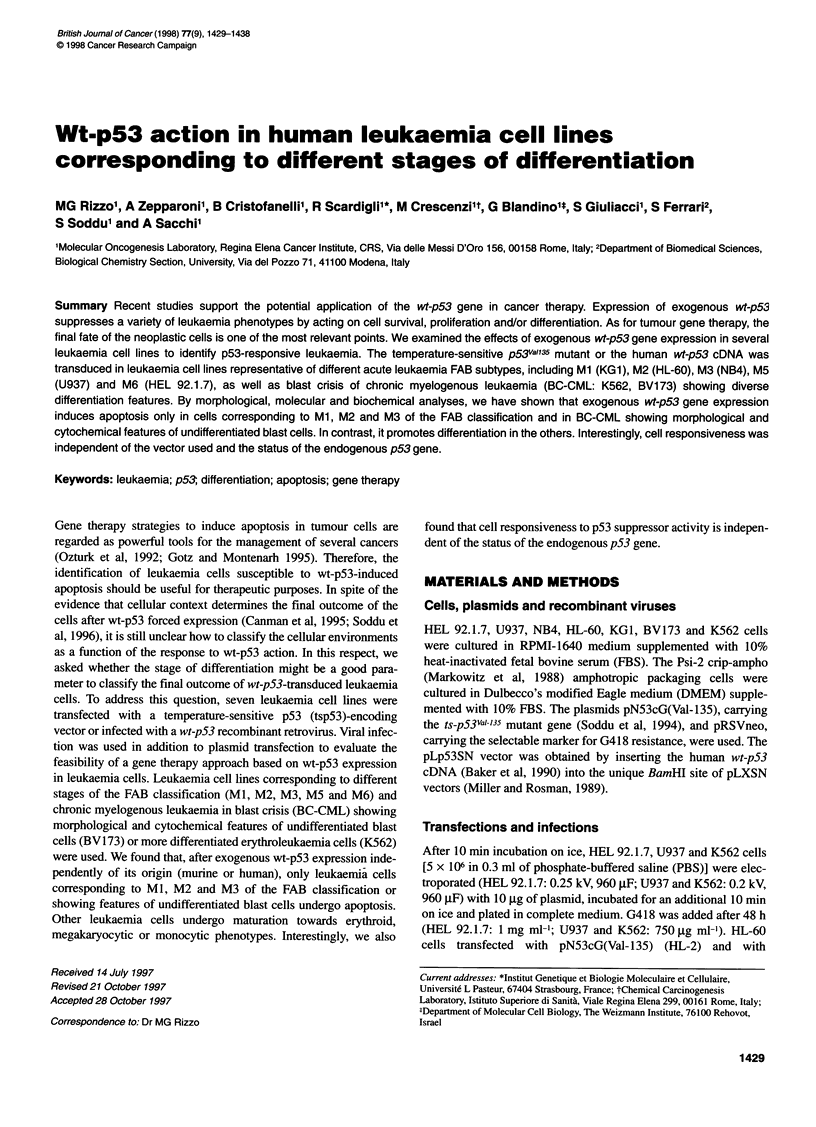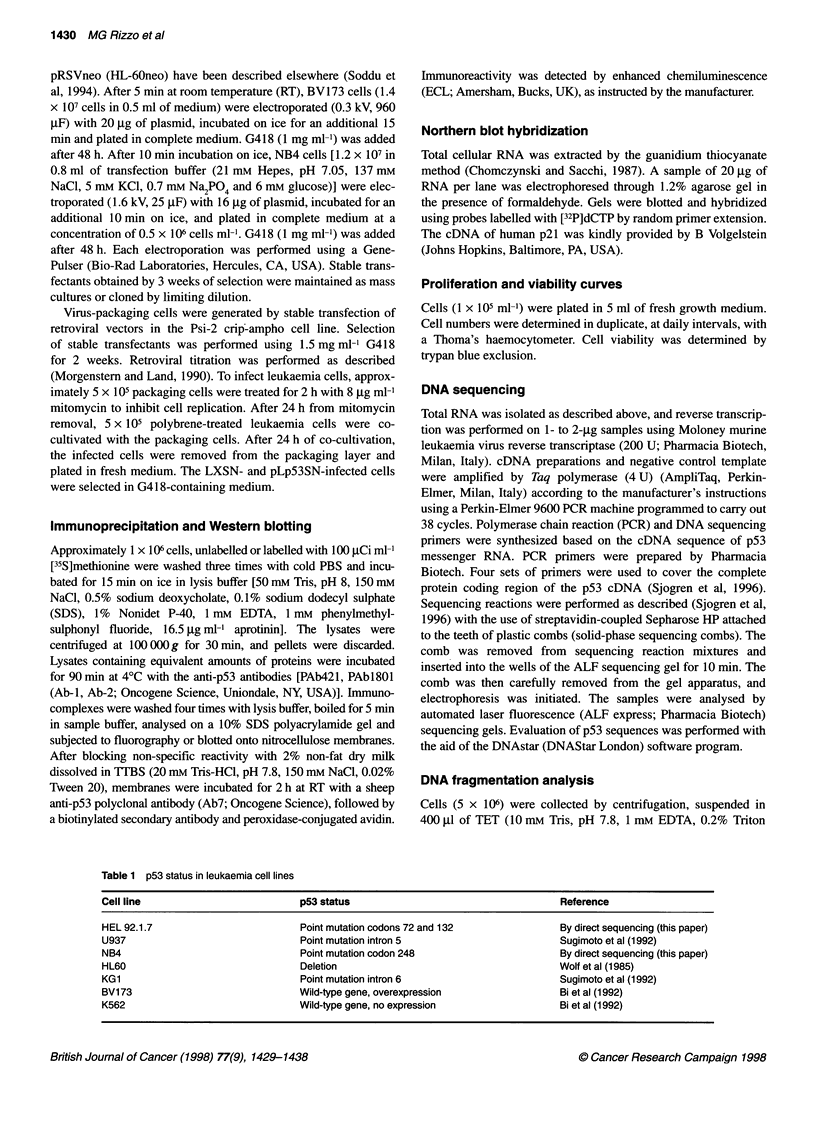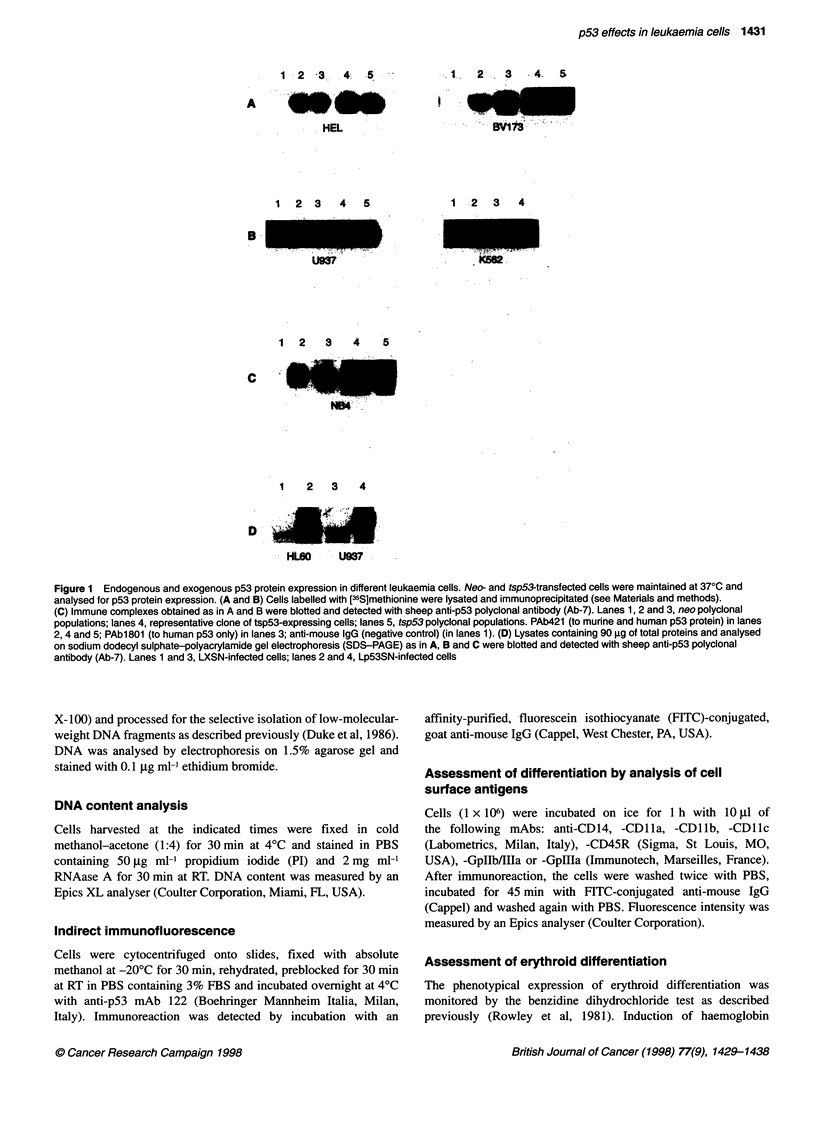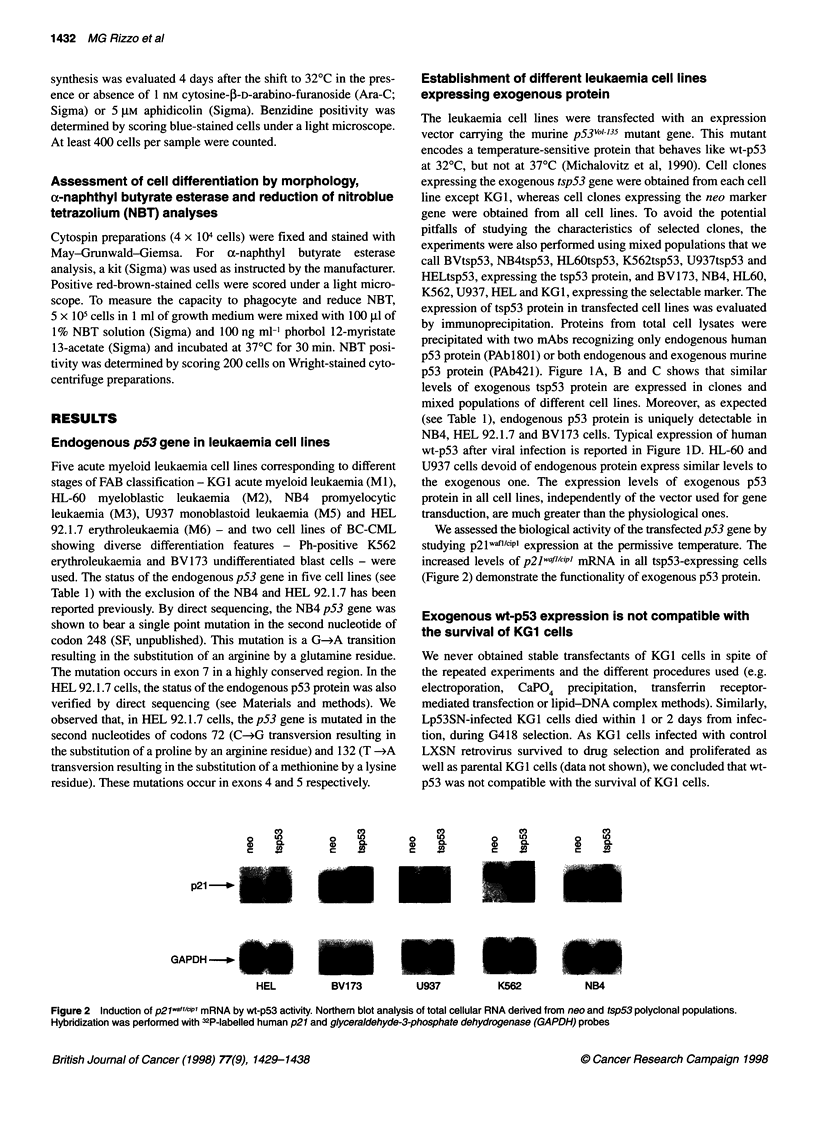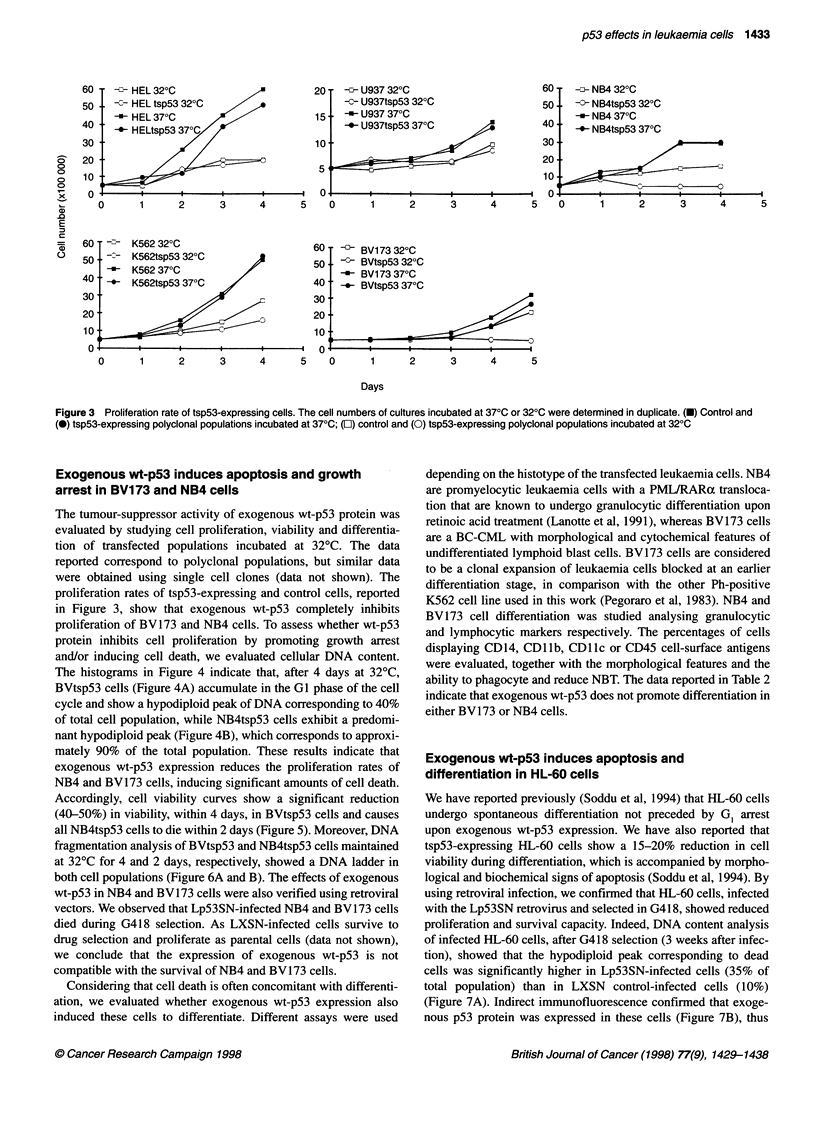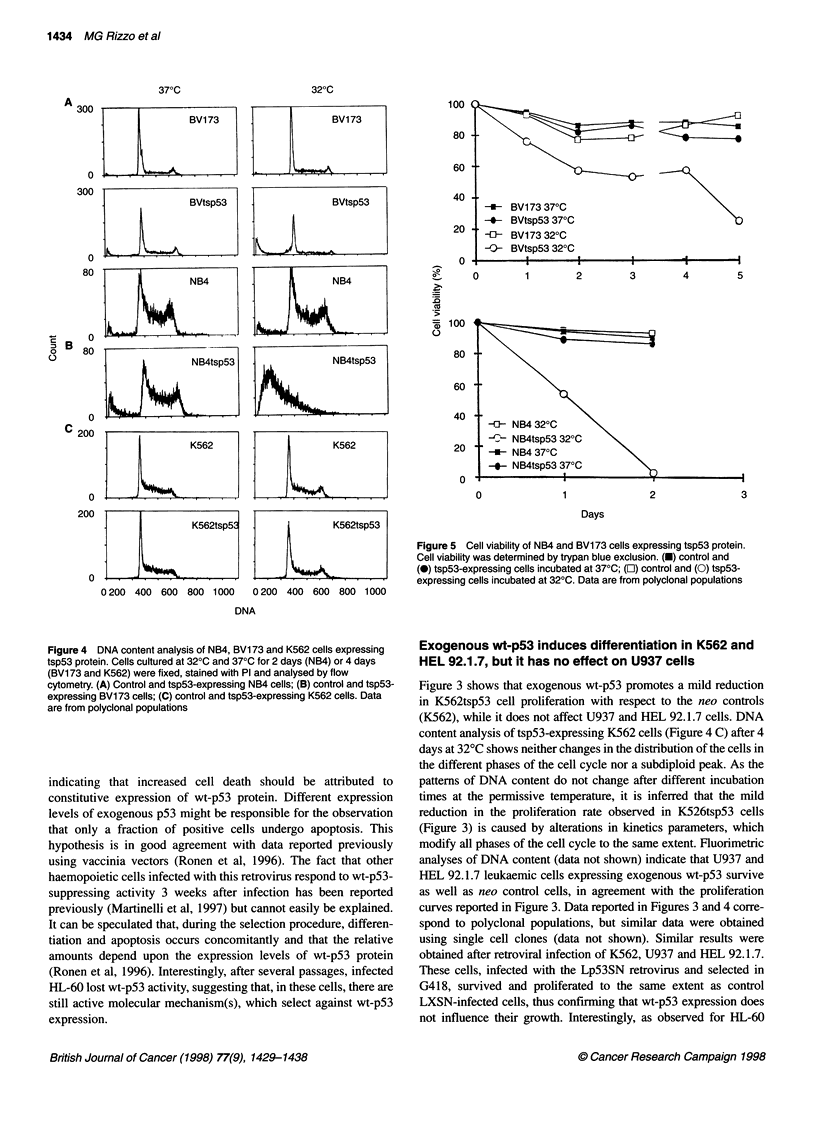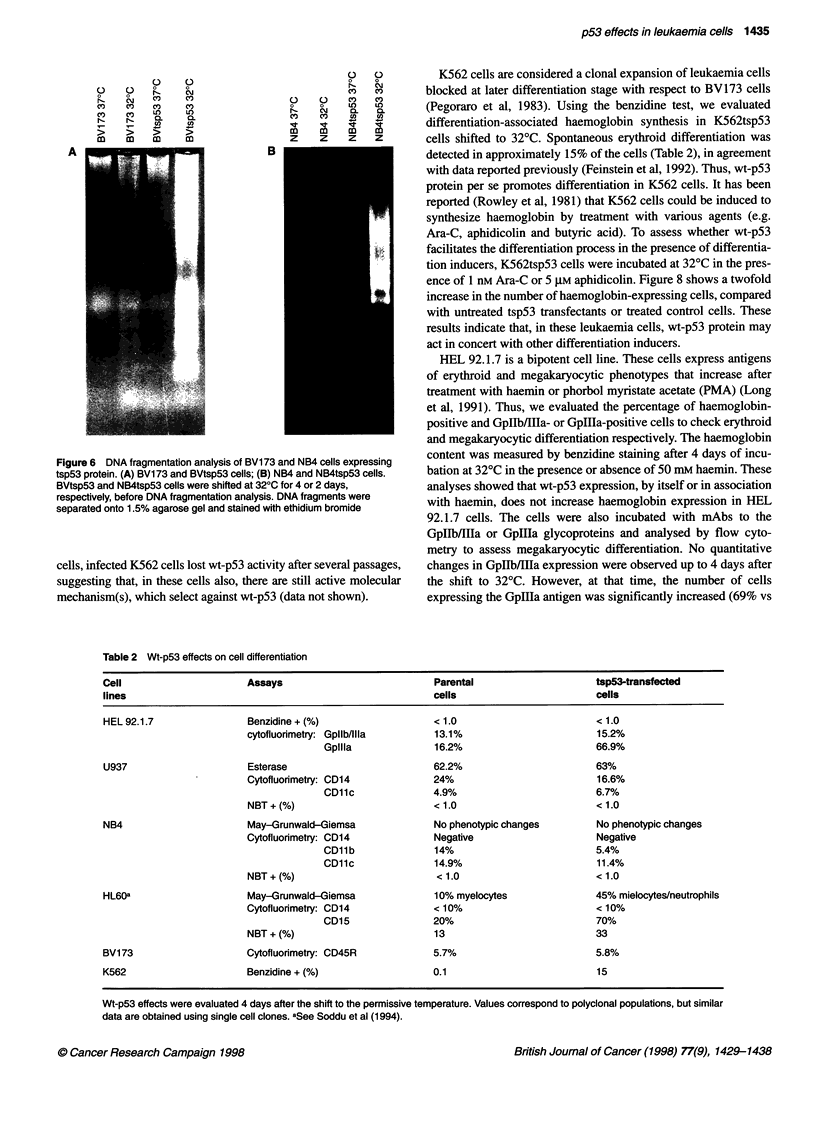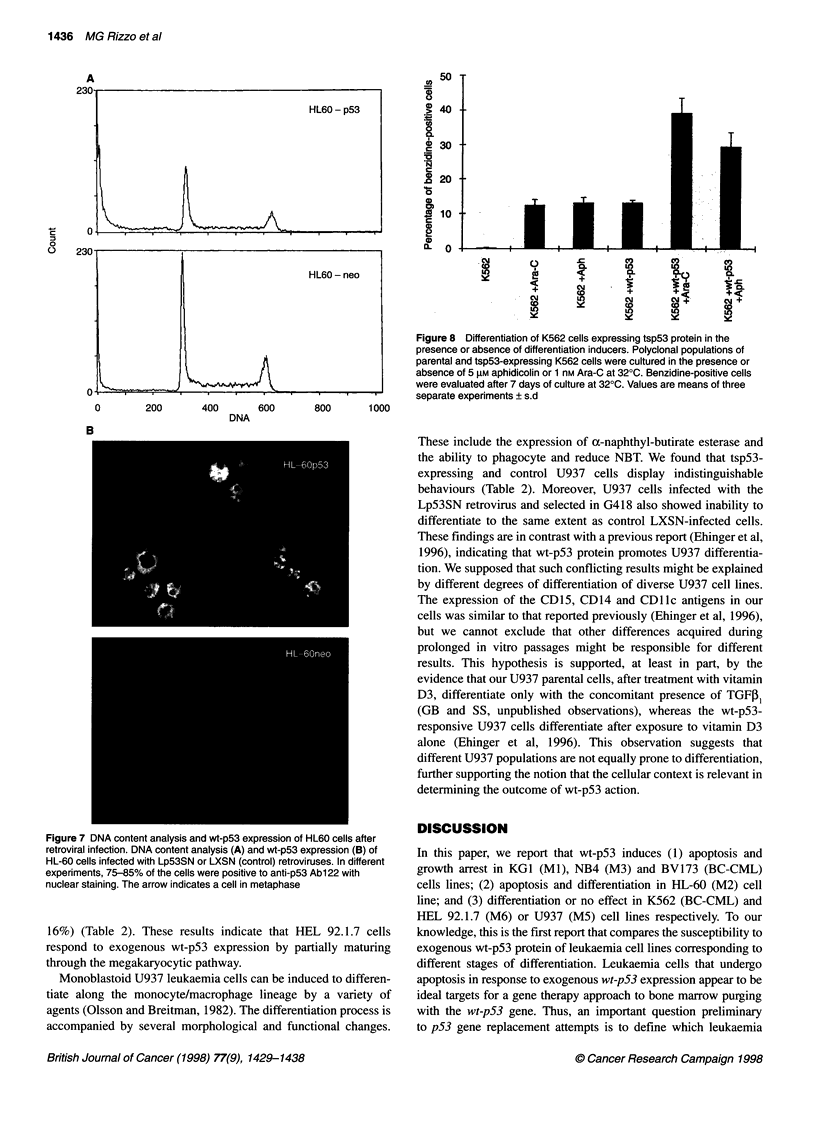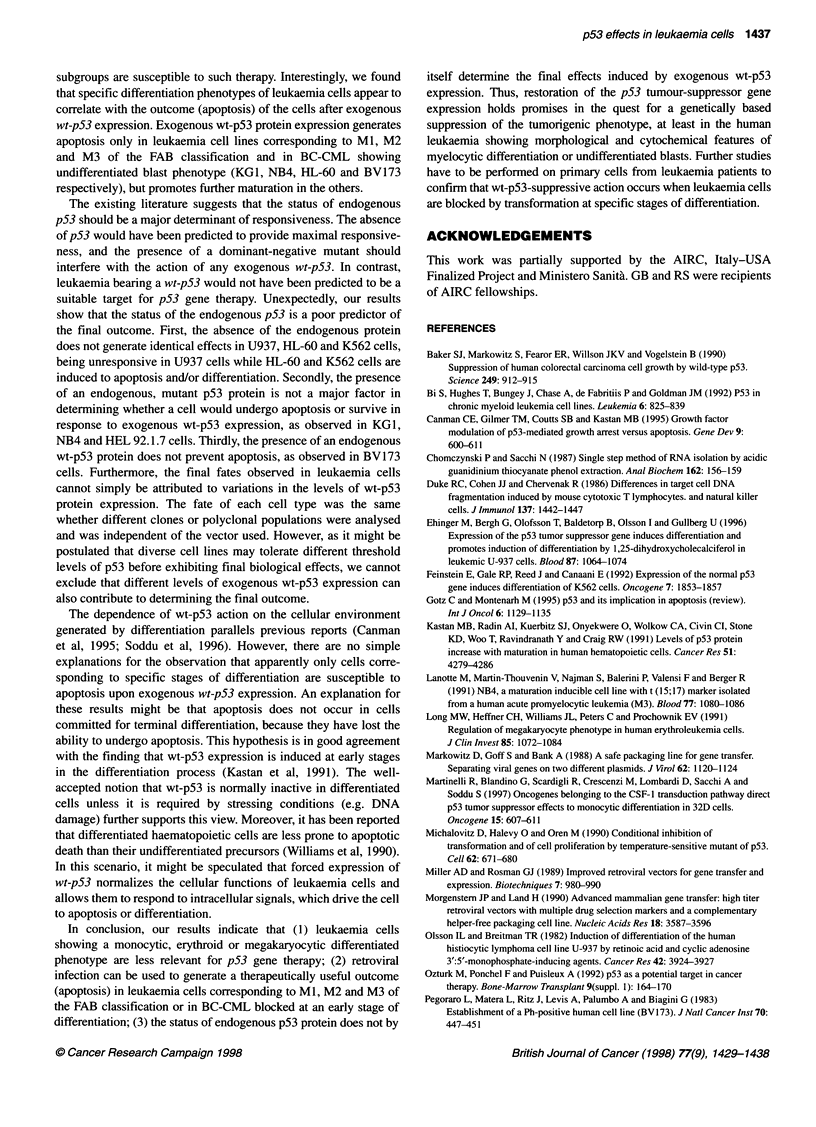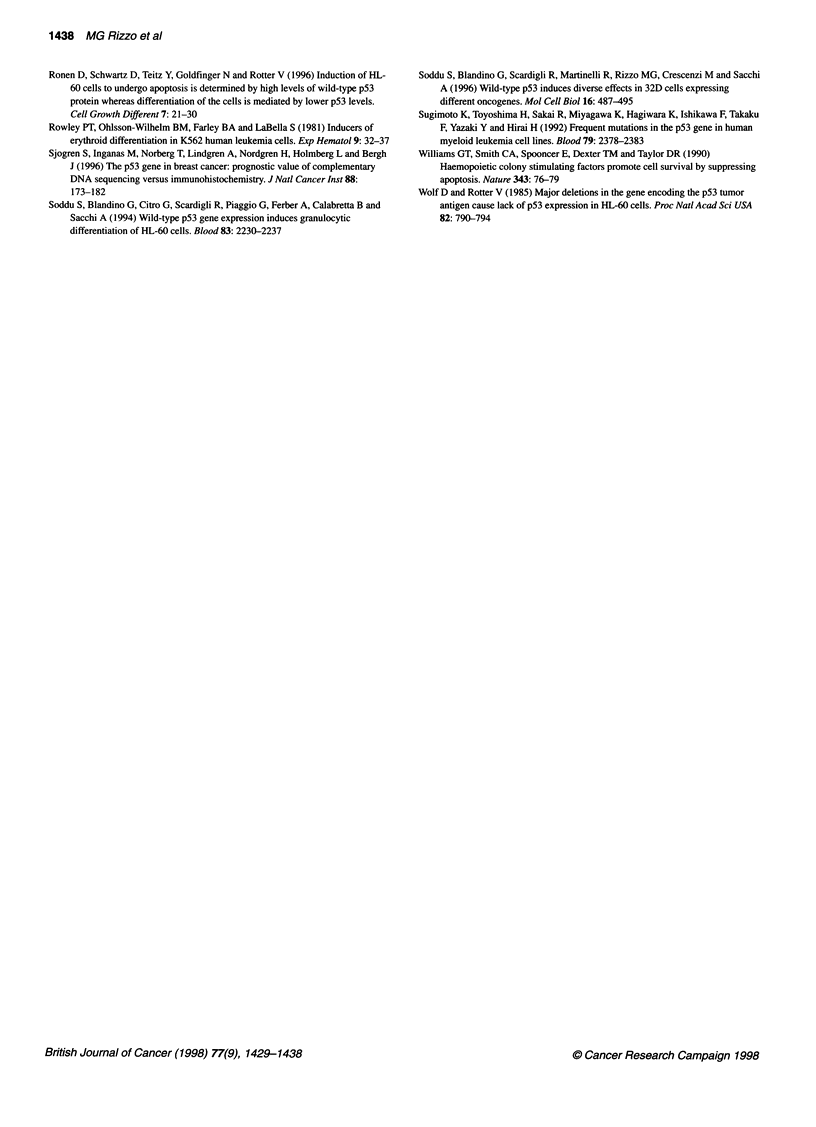# Wt-p53 action in human leukaemia cell lines corresponding to different stages of differentiation.

**DOI:** 10.1038/bjc.1998.236

**Published:** 1998-05

**Authors:** M. G. Rizzo, A. Zepparoni, B. Cristofanelli, R. Scardigli, M. Crescenzi, G. Blandino, S. Giuliacci, S. Ferrari, S. Soddu, A. Sacchi

**Affiliations:** Molecular Oncogenesis Laboratory, Regina Elena Cancer Institute, CRS, Rome, Italy.

## Abstract

**Images:**


British Journal of Cancer (1998) 77(9), 1429–1438
© 1998 Cancer Research Campaign

### Wt-p53 action in human leukaemia cell lines corresponding to different stages of differentiation

**MG Rizzo[1], A Zepparoni[1], B Cristofanelli[1], R Scardigli[1*], M Crescenzi[1†], G Blandino[1‡], S Giuliacci[1], S Ferrari[2], S Soddu[1] and A Sacchi[1]**

[1]Molecular Oncogenesis Laboratory, Regina Elena Cancer Institute, CRS, Via delle Messi D'Oro 156, 00158 Rome, Italy; [2]Department of Biomedical Sciences, Biological Chemistry Section, University, Via del Pozzo 71, 41100 Modena, Italy

**Summary** Recent studies support the potential application of the *wt-p53* gene in cancer therapy. Expression of exogenous *wt-p53* suppresses a variety of leukaemia phenotypes by acting on cell survival, proliferation and/or differentiation. As for tumour gene therapy, the final fate of the neoplastic cells is one of the most relevant points. We examined the effects of exogenous *wt-p53* gene expression in several leukaemia cell lines to identify p53-responsive leukaemia. The temperature-sensitive $p53^{Val135}$ mutant or the human *wt-p53* cDNA was transduced in leukaemia cell lines representative of different acute leukaemia FAB subtypes, including M1 (KG1), M2 (HL-60), M3 (NB4), M5 (U937) and M6 (HEL 92.1.7), as well as blast crisis of chronic myelogenous leukaemia (BC-CML: K562, BV173) showing diverse differentiation features. By morphological, molecular and biochemical analyses, we have shown that exogenous *wt-p53* gene expression induces apoptosis only in cells corresponding to M1, M2 and M3 of the FAB classification and in BC-CML showing morphological and cytochemical features of undifferentiated blast cells. In contrast, it promotes differentiation in the others. Interestingly, cell responsiveness was independent of the vector used and the status of the endogenous *p53* gene.

**Keywords:** leukaemia; *p53*; differentiation; apoptosis; gene therapy

Gene therapy strategies to induce apoptosis in tumour cells are regarded as powerful tools for the management of several cancers (Ozturk et al, 1992; Gotz and Montenarh 1995). Therefore, the identification of leukaemia cells susceptible to wt-p53-induced apoptosis should be useful for therapeutic purposes. In spite of the evidence that cellular context determines the final outcome of the cells after wt-p53 forced expression (Canman et al, 1995; Soddu et al, 1996), it is still unclear how to classify the cellular environments as a function of the response to wt-p53 action. In this respect, we asked whether the stage of differentiation might be a good parameter to classify the final outcome of *wt-p53*-transduced leukaemia cells. To address this question, seven leukaemia cell lines were transfected with a temperature-sensitive p53 (tsp53)-encoding vector or infected with a *wt-p53* recombinant retrovirus. Viral infection was used in addition to plasmid transfection to evaluate the feasibility of a gene therapy approach based on wt-p53 expression in leukaemia cells. Leukaemia cell lines corresponding to different stages of the FAB classification (M1, M2, M3, M5 and M6) and chronic myelogenous leukaemia in blast crisis (BC-CML) showing morphological and cytochemical features of undifferentiated blast cells (BV173) or more differentiated erythroleukaemia cells (K562) were used. We found that, after exogenous wt-p53 expression independently of its origin (murine or human), only leukaemia cells corresponding to M1, M2 and M3 of the FAB classification or showing features of undifferentiated blast cells undergo apoptosis. Other leukaemia cells undergo maturation towards erythroid, megakaryocytic or monocytic phenotypes. Interestingly, we also found that cell responsiveness to p53 suppressor activity is independent of the status of the endogenous *p53* gene.

*Received 14 July 1997*
*Revised 21 October 1997*
*Accepted 28 October 1997*

*Correspondence to:* Dr MG Rizzo

### MATERIALS AND METHODS

### Cells, plasmids and recombinant viruses

HEL 92.1.7, U937, NB4, HL-60, KG1, BV173 and K562 cells were cultured in RPMI-1640 medium supplemented with 10% heat-inactivated fetal bovine serum (FBS). The Psi-2 crip-ampho (Markowitz et al, 1988) amphotropic packaging cells were cultured in Dulbecco's modified Eagle medium (DMEM) supplemented with 10% FBS. The plasmids pN53cG(Val-135), carrying the $ts\text{-}p53^{Val\text{-}135}$ mutant gene (Soddu et al, 1994), and pRSVneo, carrying the selectable marker for G418 resistance, were used. The pLp53SN vector was obtained by inserting the human *wt-p53* cDNA (Baker et al, 1990) into the unique *Bam*HI site of pLXSN vectors (Miller and Rosman, 1989).

### Transfections and infections

After 10 min incubation on ice, HEL 92.1.7, U937 and K562 cells [5 × $10^6$ in 0.3 ml of phosphate-buffered saline (PBS)] were electroporated (HEL 92.1.7: 0.25 kV, 960 μF; U937 and K562: 0.2 kV, 960 μF) with 10 μg of plasmid, incubated for an additional 10 min on ice and plated in complete medium. G418 was added after 48 h (HEL 92.1.7: 1 mg $ml^{-1}$; U937 and K562: 750 μg $ml^{-1}$). HL-60 cells transfected with pN53cG(Val-135) (HL-2) and with

*Current addresses:* *Institut Genetique et Biologie Moleculaire et Cellulaire, Université L Pasteur, 67404 Strasbourg, France; †Chemical Carcinogenesis Laboratory, Istituto Superiore di Sanità, Viale Regina Elena 299, 00161 Rome, Italy; ‡Department of Molecular Cell Biology, The Weizmann Institute, 76100 Rehovot, Israel pRSVneo (HL-60neo) have been described elsewhere (Soddu et al, 1994). After 5 min at room temperature (RT), BV173 cells (1.4 × $10^7$ cells in 0.5 ml of medium) were electroporated (0.3 kV, 960 μF) with 20 μg of plasmid, incubated on ice for an additional 15 min and plated in complete medium. G418 (1 mg $ml^{-1}$) was added after 48 h. After 10 min incubation on ice, NB4 cells [1.2 × $10^7$ in 0.8 ml of transfection buffer (21 mM Hepes, pH 7.05, 137 mM NaCl, 5 mM KCl, 0.7 mM $Na_2PO_4$ and 6 mM glucose)] were electroporated (1.6 kV, 25 μF) with 16 μg of plasmid, incubated for an additional 10 min on ice, and plated in complete medium at a concentration of 0.5 × $10^6$ cells $ml^{-1}$. G418 (1 mg $ml^{-1}$) was added after 48 h. Each electroporation was performed using a Gene-Pulser (Bio-Rad Laboratories, Hercules, CA, USA). Stable transfectants obtained by 3 weeks of selection were maintained as mass cultures or cloned by limiting dilution.

Virus-packaging cells were generated by stable transfection of retroviral vectors in the Psi-2 crip-ampho cell line. Selection of stable transfectants was performed using 1.5 mg $ml^{-1}$ G418 for 2 weeks. Retroviral titration was performed as described (Morgenstern and Land, 1990). To infect leukaemia cells, approximately 5 × $10^5$ packaging cells were treated for 2 h with 8 μg $ml^{-1}$ mitomycin to inhibit cell replication. After 24 h from mitomycin removal, 5 × $10^5$ polybrene-treated leukaemia cells were co-cultivated with the packaging cells. After 24 h of co-cultivation, the infected cells were removed from the packaging layer and plated in fresh medium. The LXSN- and pLp53SN-infected cells were selected in G418-containing medium.

### Immunoprecipitation and Western blotting

Approximately 1 × $10^6$ cells, unlabelled or labelled with 100 μCi $ml^{-1}$ [$^{35}$S]methionine were washed three times with cold PBS and incubated for 15 min on ice in lysis buffer [50 mM Tris, pH 8, 150 mM NaCl, 0.5% sodium deoxycholate, 0.1% sodium dodecyl sulphate (SDS), 1% Nonidet P-40, 1 mM EDTA, 1 mM phenylmethylsulphonyl fluoride, 16.5 μg $ml^{-1}$ aprotinin]. The lysates were centrifuged at 100 000 *g* for 30 min, and pellets were discarded. Lysates containing equivalent amounts of proteins were incubated for 90 min at 4°C with the anti-p53 antibodies [PAb421, PAb1801 (Ab-1, Ab-2; Oncogene Science, Uniondale, NY, USA)]. Immunocomplexes were washed four times with lysis buffer, boiled for 5 min in sample buffer, analysed on a 10% SDS polyacrylamide gel and subjected to fluorography or blotted onto nitrocellulose membranes. After blocking non-specific reactivity with 2% non-fat dry milk dissolved in TTBS (20 mM Tris-HCl, pH 7.8, 150 mM NaCl, 0.02% Tween 20), membranes were incubated for 2 h at RT with a sheep anti-p53 polyclonal antibody (Ab7; Oncogene Science), followed by a biotinylated secondary antibody and peroxidase-conjugated avidin. Immunoreactivity was detected by enhanced chemiluminescence (ECL; Amersham, Bucks, UK), as instructed by the manufacturer.

### Northern blot hybridization

Total cellular RNA was extracted by the guanidium thiocyanate method (Chomczynski and Sacchi, 1987). A sample of 20 μg of RNA per lane was electrophoresed through 1.2% agarose gel in the presence of formaldehyde. Gels were blotted and hybridized using probes labelled with [$^{32}$P]dCTP by random primer extension. The cDNA of human p21 was kindly provided by B Volgelstein (Johns Hopkins, Baltimore, PA, USA).

### Proliferation and viability curves

Cells (1 × $10^5$ $ml^{-1}$) were plated in 5 ml of fresh growth medium. Cell numbers were determined in duplicate, at daily intervals, with a Thoma's haemocytometer. Cell viability was determined by trypan blue exclusion.

### DNA sequencing

Total RNA was isolated as described above, and reverse transcription was performed on 1- to 2-μg samples using Moloney murine leukaemia virus reverse transcriptase (200 U; Pharmacia Biotech, Milan, Italy). cDNA preparations and negative control template were amplified by *Taq* polymerase (4 U) (AmpliTaq, Perkin-Elmer, Milan, Italy) according to the manufacturer's instructions using a Perkin-Elmer 9600 PCR machine programmed to carry out 38 cycles. Polymerase chain reaction (PCR) and DNA sequencing primers were synthesized based on the cDNA sequence of p53 messenger RNA. PCR primers were prepared by Pharmacia Biotech. Four sets of primers were used to cover the complete protein coding region of the p53 cDNA (Sjogren et al, 1996). Sequencing reactions were performed as described (Sjogren et al, 1996) with the use of streptavidin-coupled Sepharose HP attached to the teeth of plastic combs (solid-phase sequencing combs). The comb was removed from sequencing reaction mixtures and inserted into the wells of the ALF sequencing gel for 10 min. The comb was then carefully removed from the gel apparatus, and electrophoresis was initiated. The samples were analysed by automated laser fluorescence (ALF express; Pharmacia Biotech) sequencing gels. Evaluation of p53 sequences was performed with the aid of the DNAstar (DNAStar London) software program.

### DNA fragmentation analysis

Cells (5 × $10^6$) were collected by centrifugation, suspended in 400 μl of TET (10 mM Tris, pH 7.8, 1 mM EDTA, 0.2% Triton

**Table 1** p53 status in leukaemia cell lines

| Cell line | p53 status | Reference |
|---|---|---|
| HEL 92.1.7 | Point mutation codons 72 and 132 | By direct sequencing (this paper) |
| U937 | Point mutation intron 5 | Sugimoto et al (1992) |
| NB4 | Point mutation codon 248 | By direct sequencing (this paper) |
| HL60 | Deletion | Wolf et al (1985) |
| KG1 | Point mutation intron 6 | Sugimoto et al (1992) |
| BV173 | Wild-type gene, overexpression | Bi et al (1992) |
| K562 | Wild-type gene, no expression | Bi et al (1992) |

© Cancer Research Campaign 1998

**Figure 1** Endogenous and exogenous p53 protein expression in different leukaemia cells. *Neo-* and *tsp53*-transfected cells were maintained at 37°C and analysed for p53 protein expression. (**A** and **B**) Cells labelled with [$^{35}$S]methionine were lysated and immunoprecipitated (see Materials and methods). (**C**) Immune complexes obtained as in A and B were blotted and detected with sheep anti-p53 polyclonal antibody (Ab-7). Lanes 1, 2 and 3, *neo* polyclonal populations; lanes 4, representative clone of tsp53-expressing cells; lanes 5, *tsp53* polyclonal populations. PAb421 (to murine and human p53 protein) in lanes 2, 4 and 5; PAb1801 (to human p53 only) in lanes 3; anti-mouse IgG (negative control) (in lanes 1). (**D**) Lysates containing 90 µg of total proteins and analysed on sodium dodecyl sulphate–polyacrylamide gel electrophoresis (SDS–PAGE) as in **A**, **B** and **C** were blotted and detected with sheep anti-p53 polyclonal antibody (Ab-7). Lanes 1 and 3, LXSN-infected cells; lanes 2 and 4, Lp53SN-infected cells

X-100) and processed for the selective isolation of low-molecular-weight DNA fragments as described previously (Duke et al, 1986). DNA was analysed by electrophoresis on 1.5% agarose gel and stained with 0.1 µg ml$^{-1}$ ethidium bromide.

### DNA content analysis

Cells harvested at the indicated times were fixed in cold methanol–acetone (1:4) for 30 min at 4°C and stained in PBS containing 50 µg ml$^{-1}$ propidium iodide (PI) and 2 mg ml$^{-1}$ RNAase A for 30 min at RT. DNA content was measured by an Epics XL analyser (Coulter Corporation, Miami, FL, USA).

### Indirect immunofluorescence

Cells were cytocentrifuged onto slides, fixed with absolute methanol at –20°C for 30 min, rehydrated, preblocked for 30 min at RT in PBS containing 3% FBS and incubated overnight at 4°C with anti-p53 mAb 122 (Boehringer Mannheim Italia, Milan, Italy). Immunoreaction was detected by incubation with an affinity-purified, fluorescein isothiocyanate (FITC)-conjugated, goat anti-mouse IgG (Cappel, West Chester, PA, USA).

### Assessment of differentiation by analysis of cell surface antigens

Cells (1 × 10$^{6}$) were incubated on ice for 1 h with 10 µl of the following mAbs: anti-CD14, -CD11a, -CD11b, -CD11c (Labometrics, Milan, Italy), -CD45R (Sigma, St Louis, MO, USA), -GpIIb/IIIa or -GpIIIa (Immunotech, Marseilles, France). After immunoreaction, the cells were washed twice with PBS, incubated for 45 min with FITC-conjugated anti-mouse IgG (Cappel) and washed again with PBS. Fluorescence intensity was measured by an Epics analyser (Coulter Corporation).

### Assessment of erythroid differentiation

The phenotypical expression of erythroid differentiation was monitored by the benzidine dihydrochloride test as described previously (Rowley et al, 1981). Induction of haemoglobin

© Cancer Research Campaign 1998

synthesis was evaluated 4 days after the shift to 32°C in the presence or absence of 1 nM cytosine-β-D-arabino-furanoside (Ara-C; Sigma) or 5 μM aphidicolin (Sigma). Benzidine positivity was determined by scoring blue-stained cells under a light microscope. At least 400 cells per sample were counted.

### Assessment of cell differentiation by morphology, α-naphthyl butyrate esterase and reduction of nitroblue tetrazolium (NBT) analyses

Cytospin preparations (4 × 10$^4$ cells) were fixed and stained with May–Grunwald–Giemsa. For α-naphthyl butyrate esterase analysis, a kit (Sigma) was used as instructed by the manufacturer. Positive red-brown-stained cells were scored under a light microscope. To measure the capacity to phagocyte and reduce NBT, 5 × 10$^5$ cells in 1 ml of growth medium were mixed with 100 μl of 1% NBT solution (Sigma) and 100 ng ml$^{-1}$ phorbol 12-myristate 13-acetate (Sigma) and incubated at 37°C for 30 min. NBT positivity was determined by scoring 200 cells on Wright-stained cytocentrifuge preparations.

### RESULTS

### Endogenous *p53* gene in leukaemia cell lines

Five acute myeloid leukaemia cell lines corresponding to different stages of FAB classification – KG1 acute myeloid leukaemia (M1), HL-60 myeloblastic leukaemia (M2), NB4 promyelocytic leukaemia (M3), U937 monoblastoid leukaemia (M5) and HEL 92.1.7 erythroleukaemia (M6) – and two cell lines of BC-CML showing diverse differentiation features – Ph-positive K562 erythroleukaemia and BV173 undifferentiated blast cells – were used. The status of the endogenous *p53* gene in five cell lines (see Table 1) with the exclusion of the NB4 and HEL 92.1.7 has been reported previously. By direct sequencing, the NB4 *p53* gene was shown to bear a single point mutation in the second nucleotide of codon 248 (SF, unpublished). This mutation is a G→A transition resulting in the substitution of an arginine by a glutamine residue. The mutation occurs in exon 7 in a highly conserved region. In the HEL 92.1.7 cells, the status of the endogenous p53 protein was also verified by direct sequencing (see Materials and methods). We observed that, in HEL 92.1.7 cells, the *p53* gene is mutated in the second nucleotides of codons 72 (C→G transversion resulting in the substitution of a proline by an arginine residue) and 132 (T →A transversion resulting in the substitution of a methionine by a lysine residue). These mutations occur in exons 4 and 5 respectively.

### Establishment of different leukaemia cell lines expressing exogenous protein

The leukaemia cell lines were transfected with an expression vector carrying the murine $p53^{Val\text{-}135}$ mutant gene. This mutant encodes a temperature-sensitive protein that behaves like wt-p53 at 32°C, but not at 37°C (Michalovitz et al, 1990). Cell clones expressing the exogenous *tsp53* gene were obtained from each cell line except KG1, whereas cell clones expressing the *neo* marker gene were obtained from all cell lines. To avoid the potential pitfalls of studying the characteristics of selected clones, the experiments were also performed using mixed populations that we call BVtsp53, NB4tsp53, HL60tsp53, K562tsp53, U937tsp53 and HELtsp53, expressing the tsp53 protein, and BV173, NB4, HL60, K562, U937, HEL and KG1, expressing the selectable marker. The expression of tsp53 protein in transfected cell lines was evaluated by immunoprecipitation. Proteins from total cell lysates were precipitated with two mAbs recognizing only endogenous human p53 protein (PAb1801) or both endogenous and exogenous murine p53 protein (PAb421). Figure 1A, B and C shows that similar levels of exogenous tsp53 protein are expressed in clones and mixed populations of different cell lines. Moreover, as expected (see Table 1), endogenous p53 protein is uniquely detectable in NB4, HEL 92.1.7 and BV173 cells. Typical expression of human wt-p53 after viral infection is reported in Figure 1D. HL-60 and U937 cells devoid of endogenous protein express similar levels to the exogenous one. The expression levels of exogenous p53 protein in all cell lines, independently of the vector used for gene transduction, are much greater than the physiological ones.

We assessed the biological activity of the transfected *p53* gene by studying $p21^{waf1/cip1}$ expression at the permissive temperature. The increased levels of $p21^{waf1/cip1}$ mRNA in all tsp53-expressing cells (Figure 2) demonstrate the functionality of exogenous p53 protein.

### Exogenous wt-p53 expression is not compatible with the survival of KG1 cells

We never obtained stable transfectants of KG1 cells in spite of the repeated experiments and the different procedures used (e.g. electroporation, $CaPO_4$ precipitation, transferrin receptor-mediated transfection or lipid–DNA complex methods). Similarly, Lp53SN-infected KG1 cells died within 1 or 2 days from infection, during G418 selection. As KG1 cells infected with control LXSN retrovirus survived to drug selection and proliferated as well as parental KG1 cells (data not shown), we concluded that wt-p53 was not compatible with the survival of KG1 cells.

**Figure 2** Induction of *p21$^{waf1/cip1}$* mRNA by wt-p53 activity. Northern blot analysis of total cellular RNA derived from *neo* and *tsp53* polyclonal populations. Hybridization was performed with $^{32}$P-labelled human *p21* and *glyceraldehyde-3-phosphate dehydrogenase (GAPDH)* probes

© Cancer Research Campaign 1998

**Figure 3** Proliferation rate of tsp53-expressing cells. The cell numbers of cultures incubated at 37°C or 32°C were determined in duplicate. (■) Control and (●) tsp53-expressing polyclonal populations incubated at 37°C; (□) control and (○) tsp53-expressing polyclonal populations incubated at 32°C

### Exogenous wt-p53 induces apoptosis and growth arrest in BV173 and NB4 cells

The tumour-suppressor activity of exogenous wt-p53 protein was evaluated by studying cell proliferation, viability and differentiation of transfected populations incubated at 32°C. The data reported correspond to polyclonal populations, but similar data were obtained using single cell clones (data not shown). The proliferation rates of tsp53-expressing and control cells, reported in Figure 3, show that exogenous wt-p53 completely inhibits proliferation of BV173 and NB4 cells. To assess whether wt-p53 protein inhibits cell proliferation by promoting growth arrest and/or inducing cell death, we evaluated cellular DNA content. The histograms in Figure 4 indicate that, after 4 days at 32°C, BVtsp53 cells (Figure 4A) accumulate in the G1 phase of the cell cycle and show a hypodiploid peak of DNA corresponding to 40% of total cell population, while NB4tsp53 cells exhibit a predominant hypodiploid peak (Figure 4B), which corresponds to approximately 90% of the total population. These results indicate that exogenous wt-p53 expression reduces the proliferation rates of NB4 and BV173 cells, inducing significant amounts of cell death. Accordingly, cell viability curves show a significant reduction (40–50%) in viability, within 4 days, in BVtsp53 cells and causes all NB4tsp53 cells to die within 2 days (Figure 5). Moreover, DNA fragmentation analysis of BVtsp53 and NB4tsp53 cells maintained at 32°C for 4 and 2 days, respectively, showed a DNA ladder in both cell populations (Figure 6A and B). The effects of exogenous wt-p53 in NB4 and BV173 cells were also verified using retroviral vectors. We observed that Lp53SN-infected NB4 and BV173 cells died during G418 selection. As LXSN-infected cells survive to drug selection and proliferate as parental cells (data not shown), we conclude that the expression of exogenous wt-p53 is not compatible with the survival of NB4 and BV173 cells.

Considering that cell death is often concomitant with differentiation, we evaluated whether exogenous wt-p53 expression also induced these cells to differentiate. Different assays were used depending on the histotype of the transfected leukaemia cells. NB4 are promyelocytic leukaemia cells with a PML/RARα translocation that are known to undergo granulocytic differentiation upon retinoic acid treatment (Lanotte et al, 1991), whereas BV173 cells are a BC-CML with morphological and cytochemical features of undifferentiated lymphoid blast cells. BV173 cells are considered to be a clonal expansion of leukaemia cells blocked at an earlier differentiation stage, in comparison with the other Ph-positive K562 cell line used in this work (Pegoraro et al, 1983). NB4 and BV173 cell differentiation was studied analysing granulocytic and lymphocytic markers respectively. The percentages of cells displaying CD14, CD11b, CD11c or CD45 cell-surface antigens were evaluated, together with the morphological features and the ability to phagocyte and reduce NBT. The data reported in Table 2 indicate that exogenous wt-p53 does not promote differentiation in either BV173 or NB4 cells.

### Exogenous wt-p53 induces apoptosis and differentiation in HL-60 cells

We have reported previously (Soddu et al, 1994) that HL-60 cells undergo spontaneous differentiation not preceded by $G_1$ arrest upon exogenous wt-p53 expression. We have also reported that tsp53-expressing HL-60 cells show a 15–20% reduction in cell viability during differentiation, which is accompanied by morphological and biochemical signs of apoptosis (Soddu et al, 1994). By using retroviral infection, we confirmed that HL-60 cells, infected with the Lp53SN retrovirus and selected in G418, showed reduced proliferation and survival capacity. Indeed, DNA content analysis of infected HL-60 cells, after G418 selection (3 weeks after infection), showed that the hypodiploid peak corresponding to dead cells was significantly higher in Lp53SN-infected cells (35% of total population) than in LXSN control-infected cells (10%) (Figure 7A). Indirect immunofluorescence confirmed that exogenous p53 protein was expressed in these cells (Figure 7B), thus

© Cancer Research Campaign 1998

Figure 4 DNA content analysis of NB4, BV173 and K562 cells expressing tsp53 protein. Cells cultured at 32°C and 37°C for 2 days (NB4) or 4 days (BV173 and K562) were fixed, stained with PI and analysed by flow cytometry. (A) Control and tsp53-expressing NB4 cells; (B) control and tsp53-expressing BV173 cells; (C) control and tsp53-expressing K562 cells. Data are from polyclonal populations

Figure 5 Cell viability of NB4 and BV173 cells expressing tsp53 protein. Cell viability was determined by trypan blue exclusion. (■) control and (●) tsp53-expressing cells incubated at 37°C; (□) control and (○) tsp53-expressing cells incubated at 32°C. Data are from polyclonal populations indicating that increased cell death should be attributed to constitutive expression of wt-p53 protein. Different expression levels of exogenous p53 might be responsible for the observation that only a fraction of positive cells undergo apoptosis. This hypothesis is in good agreement with data reported previously using vaccinia vectors (Ronen et al, 1996). The fact that other haemopoietic cells infected with this retrovirus respond to wt-p53-suppressing activity 3 weeks after infection has been reported previously (Martinelli et al, 1997) but cannot easily be explained. It can be speculated that, during the selection procedure, differentiation and apoptosis occurs concomitantly and that the relative amounts depend upon the expression levels of wt-p53 protein (Ronen et al, 1996). Interestingly, after several passages, infected HL-60 lost wt-p53 activity, suggesting that, in these cells, there are still active molecular mechanism(s), which select against wt-p53 expression.

### Exogenous wt-p53 induces differentiation in K562 and HEL 92.1.7, but it has no effect on U937 cells

Figure 3 shows that exogenous wt-p53 promotes a mild reduction in K562tsp53 cell proliferation with respect to the *neo* controls (K562), while it does not affect U937 and HEL 92.1.7 cells. DNA content analysis of tsp53-expressing K562 cells (Figure 4 C) after 4 days at 32°C shows neither changes in the distribution of the cells in the different phases of the cell cycle nor a subdiploid peak. As the patterns of DNA content do not change after different incubation times at the permissive temperature, it is inferred that the mild reduction in the proliferation rate observed in K526tsp53 cells (Figure 3) is caused by alterations in kinetics parameters, which modify all phases of the cell cycle to the same extent. Fluorimetric analyses of DNA content (data not shown) indicate that U937 and HEL 92.1.7 leukaemic cells expressing exogenous wt-p53 survive as well as *neo* control cells, in agreement with the proliferation curves reported in Figure 3. Data reported in Figures 3 and 4 correspond to polyclonal populations, but similar data were obtained using single cell clones (data not shown). Similar results were obtained after retroviral infection of K562, U937 and HEL 92.1.7. These cells, infected with the Lp53SN retrovirus and selected in G418, survived and proliferated to the same extent as control LXSN-infected cells, thus confirming that wt-p53 expression does not influence their growth. Interestingly, as observed for HL-60

© Cancer Research Campaign 1998

**Figure 6** DNA fragmentation analysis of BV173 and NB4 cells expressing tsp53 protein. (**A**) BV173 and BVtsp53 cells; (**B**) NB4 and NB4tsp53 cells. BVtsp53 and NB4tsp53 cells were shifted at 32°C for 4 or 2 days, respectively, before DNA fragmentation analysis. DNA fragments were separated onto 1.5% agarose gel and stained with ethidium bromide cells, infected K562 cells lost wt-p53 activity after several passages, suggesting that, in these cells also, there are still active molecular mechanism(s), which select against wt-p53 (data not shown).

K562 cells are considered a clonal expansion of leukaemia cells blocked at later differentiation stage with respect to BV173 cells (Pegoraro et al, 1983). Using the benzidine test, we evaluated differentiation-associated haemoglobin synthesis in K562tsp53 cells shifted to 32°C. Spontaneous erythroid differentiation was detected in approximately 15% of the cells (Table 2), in agreement with data reported previously (Feinstein et al, 1992). Thus, wt-p53 protein per se promotes differentiation in K562 cells. It has been reported (Rowley et al, 1981) that K562 cells could be induced to synthesize haemoglobin by treatment with various agents (e.g. Ara-C, aphidicolin and butyric acid). To assess whether wt-p53 facilitates the differentiation process in the presence of differentiation inducers, K562tsp53 cells were incubated at 32°C in the presence of 1 nM Ara-C or 5 µM aphidicolin. Figure 8 shows a twofold increase in the number of haemoglobin-expressing cells, compared with untreated tsp53 transfectants or treated control cells. These results indicate that, in these leukaemia cells, wt-p53 protein may act in concert with other differentiation inducers.

HEL 92.1.7 is a bipotent cell line. These cells express antigens of erythroid and megakaryocytic phenotypes that increase after treatment with haemin or phorbol myristate acetate (PMA) (Long et al, 1991). Thus, we evaluated the percentage of haemoglobin-positive and GpIIb/IIIa- or GpIIIa-positive cells to check erythroid and megakaryocytic differentiation respectively. The haemoglobin content was measured by benzidine staining after 4 days of incubation at 32°C in the presence or absence of 50 mM haemin. These analyses showed that wt-p53 expression, by itself or in association with haemin, does not increase haemoglobin expression in HEL 92.1.7 cells. The cells were also incubated with mAbs to the GpIIb/IIIa or GpIIIa glycoproteins and analysed by flow cytometry to assess megakaryocytic differentiation. No quantitative changes in GpIIb/IIIa expression were observed up to 4 days after the shift to 32°C. However, at that time, the number of cells expressing the GpIIIa antigen was significantly increased (69% vs

**Table 2** Wt-p53 effects on cell differentiation

| Cell lines | Assays | Parental cells | tsp53-transfected cells |
|---|---|---|---|
| HEL 92.1.7 | Benzidine + (%) | < 1.0 | < 1.0 |
| | cytofluorimetry: GpIIb/IIIa | 13.1% | 15.2% |
| | GpIIIa | 16.2% | 66.9% |
| U937 | Esterase | 62.2% | 63% |
| | Cytofluorimetry: CD14 | 24% | 16.6% |
| | CD11c | 4.9% | 6.7% |
| | NBT + (%) | < 1.0 | < 1.0 |
| NB4 | May–Grunwald–Giemsa | No phenotypic changes | No phenotypic changes |
| | Cytofluorimetry: CD14 | Negative | Negative |
| | CD11b | 14% | 5.4% |
| | CD11c | 14.9% | 11.4% |
| | NBT + (%) | < 1.0 | < 1.0 |
| HL60[a] | May–Grunwald–Giemsa | 10% myelocytes | 45% mielocytes/neutrophils |
| | Cytofluorimetry: CD14 | < 10% | < 10% |
| | CD15 | 20% | 70% |
| | NBT + (%) | 13 | 33 |
| BV173 | Cytofluorimetry: CD45R | 5.7% | 5.8% |
| K562 | Benzidine + (%) | 0.1 | 15 |

Wt-p53 effects were evaluated 4 days after the shift to the permissive temperature. Values correspond to polyclonal populations, but similar data are obtained using single cell clones. [a]See Soddu et al (1994).

© Cancer Research Campaign 1998 

**Figure 7** DNA content analysis and wt-p53 expression of HL60 cells after retroviral infection. DNA content analysis (**A**) and wt-p53 expression (**B**) of HL-60 cells infected with Lp53SN or LXSN (control) retroviruses. In different experiments, 75–85% of the cells were positive to anti-p53 Ab122 with nuclear staining. The arrow indicates a cell in metaphase

**Figure 8** Differentiation of K562 cells expressing tsp53 protein in the presence or absence of differentiation inducers. Polyclonal populations of parental and tsp53-expressing K562 cells were cultured in the presence or absence of 5 μM aphidicolin or 1 nM Ara-C at 32°C. Benzidine-positive cells were evaluated after 7 days of culture at 32°C. Values are means of three separate experiments ± s.d

16%) (Table 2). These results indicate that HEL 92.1.7 cells respond to exogenous wt-p53 expression by partially maturing through the megakaryocytic pathway.

Monoblastoid U937 leukaemia cells can be induced to differentiate along the monocyte/macrophage lineage by a variety of agents (Olsson and Breitman, 1982). The differentiation process is accompanied by several morphological and functional changes. These include the expression of α-naphthyl-butirate esterase and the ability to phagocyte and reduce NBT. We found that tsp53-expressing and control U937 cells display indistinguishable behaviours (Table 2). Moreover, U937 cells infected with the Lp53SN retrovirus and selected in G418 also showed inability to differentiate to the same extent as control LXSN-infected cells. These findings are in contrast with a previous report (Ehinger et al, 1996), indicating that wt-p53 protein promotes U937 differentiation. We supposed that such conflicting results might be explained by different degrees of differentiation of diverse U937 cell lines. The expression of the CD15, CD14 and CD11c antigens in our cells was similar to that reported previously (Ehinger et al, 1996), but we cannot exclude that other differences acquired during prolonged in vitro passages might be responsible for different results. This hypothesis is supported, at least in part, by the evidence that our U937 parental cells, after treatment with vitamin D3, differentiate only with the concomitant presence of $TGF\beta_1$ (GB and SS, unpublished observations), whereas the wt-p53-responsive U937 cells differentiate after exposure to vitamin D3 alone (Ehinger et al, 1996). This observation suggests that different U937 populations are not equally prone to differentiation, further supporting the notion that the cellular context is relevant in determining the outcome of wt-p53 action.

### DISCUSSION

In this paper, we report that wt-p53 induces (1) apoptosis and growth arrest in KG1 (M1), NB4 (M3) and BV173 (BC-CML) cells lines; (2) apoptosis and differentiation in HL-60 (M2) cell line; and (3) differentiation or no effect in K562 (BC-CML) and HEL 92.1.7 (M6) or U937 (M5) cell lines respectively. To our knowledge, this is the first report that compares the susceptibility to exogenous wt-p53 protein of leukaemia cell lines corresponding to different stages of differentiation. Leukaemia cells that undergo apoptosis in response to exogenous *wt-p53* expression appear to be ideal targets for a gene therapy approach to bone marrow purging with the *wt-p53* gene. Thus, an important question preliminary to *p53* gene replacement attempts is to define which leukaemia

© Cancer Research Campaign 1998

subgroups are susceptible to such therapy. Interestingly, we found that specific differentiation phenotypes of leukaemia cells appear to correlate with the outcome (apoptosis) of the cells after exogenous *wt-p53* expression. Exogenous wt-p53 protein expression generates apoptosis only in leukaemia cell lines corresponding to M1, M2 and M3 of the FAB classification and in BC-CML showing undifferentiated blast phenotype (KG1, NB4, HL-60 and BV173 respectively), but promotes further maturation in the others.

The existing literature suggests that the status of endogenous *p53* should be a major determinant of responsiveness. The absence of *p53* would have been predicted to provide maximal responsiveness, and the presence of a dominant-negative mutant should interfere with the action of any exogenous *wt-p53*. In contrast, leukaemia bearing a *wt-p53* would not have been predicted to be a suitable target for *p53* gene therapy. Unexpectedly, our results show that the status of the endogenous *p53* is a poor predictor of the final outcome. First, the absence of the endogenous protein does not generate identical effects in U937, HL-60 and K562 cells, being unresponsive in U937 cells while HL-60 and K562 cells are induced to apoptosis and/or differentiation. Secondly, the presence of an endogenous, mutant p53 protein is not a major factor in determining whether a cell would undergo apoptosis or survive in response to exogenous wt-p53 expression, as observed in KG1, NB4 and HEL 92.1.7 cells. Thirdly, the presence of an endogenous wt-p53 protein does not prevent apoptosis, as observed in BV173 cells. Furthermore, the final fates observed in leukaemia cells cannot simply be attributed to variations in the levels of wt-p53 protein expression. The fate of each cell type was the same whether different clones or polyclonal populations were analysed and was independent of the vector used. However, as it might be postulated that diverse cell lines may tolerate different threshold levels of p53 before exhibiting final biological effects, we cannot exclude that different levels of exogenous wt-p53 expression can also contribute to determining the final outcome.

The dependence of wt-p53 action on the cellular environment generated by differentiation parallels previous reports (Canman et al, 1995; Soddu et al, 1996). However, there are no simple explanations for the observation that apparently only cells corresponding to specific stages of differentiation are susceptible to apoptosis upon exogenous *wt-p53* expression. An explanation for these results might be that apoptosis does not occur in cells committed for terminal differentiation, because they have lost the ability to undergo apoptosis. This hypothesis is in good agreement with the finding that wt-p53 expression is induced at early stages in the differentiation process (Kastan et al, 1991). The well-accepted notion that wt-p53 is normally inactive in differentiated cells unless it is required by stressing conditions (e.g. DNA damage) further supports this view. Moreover, it has been reported that differentiated haematopoietic cells are less prone to apoptotic death than their undifferentiated precursors (Williams et al, 1990). In this scenario, it might be speculated that forced expression of *wt-p53* normalizes the cellular functions of leukaemia cells and allows them to respond to intracellular signals, which drive the cell to apoptosis or differentiation.

In conclusion, our results indicate that (1) leukaemia cells showing a monocytic, erythroid or megakaryocytic differentiated phenotype are less relevant for *p53* gene therapy; (2) retroviral infection can be used to generate a therapeutically useful outcome (apoptosis) in leukaemia cells corresponding to M1, M2 and M3 of the FAB classification or in BC-CML blocked at an early stage of differentiation; (3) the status of endogenous p53 protein does not by itself determine the final effects induced by exogenous wt-p53 expression. Thus, restoration of the *p53* tumour-suppressor gene expression holds promises in the quest for a genetically based suppression of the tumorigenic phenotype, at least in the human leukaemia showing morphological and cytochemical features of myelocytic differentiation or undifferentiated blasts. Further studies have to be performed on primary cells from leukaemia patients to confirm that wt-p53-suppressive action occurs when leukaemia cells are blocked by transformation at specific stages of differentiation.

### ACKNOWLEDGEMENTS

This work was partially supported by the AIRC, Italy–USA Finalized Project and Ministero Sanità. GB and RS were recipients of AIRC fellowships.

### REFERENCES

Baker SJ, Markowitz S, Fearor ER, Willson JKV and Vogelstein B (1990) Suppression of human colorectal carcinoma cell growth by wild-type p53. *Science* **249**: 912–915

Bi S, Hughes T, Bungey J, Chase A, de Fabritiis P and Goldman JM (1992) P53 in chronic myeloid leukemia cell lines. *Leukemia* **6**: 825–839

Canman CE, Gilmer TM, Coutts SB and Kastan MB (1995) Growth factor modulation of p53-mediated growth arrest versus apoptosis. *Gene Dev* **9**: 600–611

Chomczynski P and Sacchi N (1987) Single step method of RNA isolation by acidic guanidinium thiocyanate phenol extraction. *Anal Biochem* **162**: 156–159

Duke RC, Cohen JJ and Chervenak R (1986) Differences in target cell DNA fragmentation induced by mouse cytotoxic T lymphocytes. and natural killer cells. *J Immunol* **137**: 1442–1447

Ehinger M, Bergh G, Olofsson T, Baldetorp B, Olsson I and Gullberg U (1996) Expression of the p53 tumor suppressor gene induces differentiation and promotes induction of differentiation by 1,25-dihydroxycholecalciferol in leukemic U-937 cells. *Blood* **87**: 1064–1074

Feinstein E, Gale RP, Reed J and Canaani E (1992) Expression of the normal p53 gene induces differentiation of K562 cells. *Oncogene* **7**: 1853–1857

Gotz C and Montenarh M (1995) p53 and its implication in apoptosis (review). *Int J Oncol* **6**: 1129–1135

Kastan MB, Radin AI, Kuerbitz SJ, Onyekwere O, Wolkow CA, Civin CI, Stone KD, Woo T, Ravindranath Y and Craig RW (1991) Levels of p53 protein increase with maturation in human hematopoietic cells. *Cancer Res* **51**: 4279–4286

Lanotte M, Martin-Thouvenin V, Najman S, Balerini P, Valensi F and Berger R (1991) NB4, a maturation inducible cell line with t (15;17) marker isolated from a human acute promyelocytic leukemia (M3). *Blood* **77**: 1080–1086

Long MW, Heffner CH, Williams JL, Peters C and Prochownik EV (1991) Regulation of megakaryocyte phenotype in human erythroleukemia cells. *J Clin Invest* **85**: 1072–1084

Markowitz D, Goff S and Bank A (1988) A safe packaging line for gene transfer. Separating viral genes on two different plasmids. *J Virol* **62**: 1120–1124

Martinelli R, Blandino G, Scardigli R, Crescenzi M, Lombardi D, Sacchi A and Soddu S (1997) Oncogenes belonging to the CSF-1 transduction pathway direct p53 tumor suppressor effects to monocytic differentiation in 32D cells. *Oncogene* **15**: 607–611

Michalovitz D, Halevy O and Oren M (1990) Conditional inhibition of transformation and of cell proliferation by temperature-sensitive mutant of p53. *Cell* **62**: 671–680

Miller AD and Rosman GJ (1989) Improved retroviral vectors for gene transfer and expression. *Biotechniques* **7**: 980–990

Morgenstern JP and Land H (1990) Advanced mammalian gene transfer: high titer retroviral vectors with multiple drug selection markers and a complementary helper-free packaging cell line. *Nucleic Acids Res* **18**: 3587–3596

Olsson IL and Breitman TR (1982) Induction of differentiation of the human histiocytic lymphoma cell line U-937 by retinoic acid and cyclic adenosine 3′:5′-monophosphate-inducing agents. *Cancer Res* **42**: 3924–3927

Ozturk M, Ponchel F and Puisleux A (1992) p53 as a potential target in cancer therapy. *Bone-Marrow Transplant* **9**(suppl. 1): 164–170

Pegoraro L, Matera L, Ritz J, Levis A, Palumbo A and Biagini G (1983) Establishment of a Ph-positive human cell line (BV173). *J Natl Cancer Inst* **70**: 447–451

© Cancer Research Campaign 1998

Ronen D, Schwartz D, Teitz Y, Goldfinger N and Rotter V (1996) Induction of HL-60 cells to undergo apoptosis is determined by high levels of wild-type p53 protein whereas differentiation of the cells is mediated by lower p53 levels. *Cell Growth Different* **7**: 21–30

Rowley PT, Ohlsson-Wilhelm BM, Farley BA and LaBella S (1981) Inducers of erythroid differentiation in K562 human leukemia cells. *Exp Hematol* **9**: 32–37

Sjogren S, Inganas M, Norberg T, Lindgren A, Nordgren H, Holmberg L and Bergh J (1996) The p53 gene in breast cancer: prognostic value of complementary DNA sequencing versus immunohistochemistry. *J Natl Cancer Inst* **88**: 173–182

Soddu S, Blandino G, Citro G, Scardigli R, Piaggio G, Ferber A, Calabretta B and Sacchi A (1994) Wild-type p53 gene expression induces granulocytic differentiation of HL-60 cells. *Blood* **83**: 2230–2237

Soddu S, Blandino G, Scardigli R, Martinelli R, Rizzo MG, Crescenzi M and Sacchi A (1996) Wild-type p53 induces diverse effects in 32D cells expressing different oncogenes. *Mol Cell Biol* **16**: 487–495

Sugimoto K, Toyoshima H, Sakai R, Miyagawa K, Hagiwara K, Ishikawa F, Takaku F, Yazaki Y and Hirai H (1992) Frequent mutations in the p53 gene in human myeloid leukemia cell lines. *Blood* **79**: 2378–2383

Williams GT, Smith CA, Spooncer E, Dexter TM and Taylor DR (1990) Haemopoietic colony stimulating factors promote cell survival by suppressing apoptosis. *Nature* **343**: 76–79

Wolf D and Rotter V (1985) Major deletions in the gene encoding the p53 tumor antigen cause lack of p53 expression in HL-60 cells. *Proc Natl Acad Sci USA* **82**: 790–794

© Cancer Research Campaign 1998